# The Effect of pH on the Structure and Lateral Organization of Cardiolipin in Langmuir Monolayers

**DOI:** 10.1002/cphc.202200218

**Published:** 2022-08-03

**Authors:** Marina Sturm, Olof Gutowski, Gerald Brezesinski

**Affiliations:** ^1^ Photon Science Deutsches Elektronen-Synchrotron DESY Notkestr. 85 22607 Hamburg Germany; ^2^ Institute of Applied Dermatopharmacy Martin Luther University Halle-Wittenberg Weinbergweg 23 06120 Halle (Saale) Germany

**Keywords:** cardiolipin, Langmuir monolayers, pH dependency, GIXD, X-ray fluorescence

## Abstract

Cardiolipin (CL) is a unique phospholipid featuring a dimeric structure. With its four alkyl chains, it has a large hydrophobic region and the charged hydrophilic head group is relatively small. Biological membranes exhibit CL exclusively in the inner bacterial and mitochondrial membranes. Alteration of CL packing can lead to structural changes and membrane instabilities. One environmental influence is the change in pH. Since the acidic properties of the phosphate head groups remain still controversial in literature, this work focusses on the influence of pH on the ionization degree of CL. For the analyses, surface pressure (π) – molecular area (A) isotherm experiments were combined with total reflection X‐ray fluorescence (TRXF) and grazing incidence X‐ray diffraction (GIXD). Continuous ionization with a high CL packing density was observed in the monolayer over a wide pH range. No individual pKa values can be assigned to the two phosphate groups, but mutual influence is observed.

## Graphical abstract

**The structures and ionization behavior** of cardiolipin (CL) with saturated chains have been investigated in model monolayers since CL plays an important role in the structural organization of biological membranes. Traditional pressure/area isotherms combined with total reflection X‐ray fluorescence (TRXF) and grazing incidence X‐ray diffraction (GIXD) elucidate the properties of CL over a wide pH range.

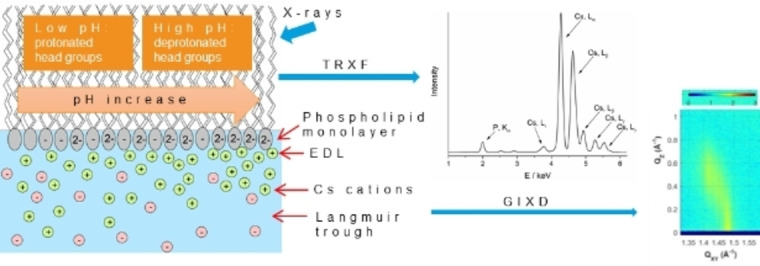

## Introduction

The anionic cardiolipin (CL) is an important phospholipid found in the plasma membranes of many types of Gram‐negative and Gram‐positive bacteria, and in the mitochondrial and chloroplast inner membranes of eukaryotes. Mitochondrial membranes have a high CL content in the inner membrane, where it contributes for up to 20 % of total lipids.^[1]^ Several signaling pathways are directly dependent on CL for their activation.^[2]^ CL plays an important role in the structural stabilization and activation of many mitochondrial enzymes.^[3]^ Additionally, CL is important for maintaining the full functionality of mitochondrial membranes through administering their structural organization.[^4^, ^5^] It has a protective function against environmental stresses.^[6]^ The composition of mitochondrial CLs depends strongly on the lipid environment in cultured cells and favors the incorporation of linoleic acid over other fatty acids.^[7]^ The presence of unsaturated fatty acids in CL might be the main reason for its susceptibility to oxidative damage. The most abundant CL species from various organisms and tissues contain only one or two types of fatty acids, resulting in a high degree of structural uniformity and molecular symmetry.^[8]^ Although the percentage of saturated fatty alkyl side chains in CL is low, the use of such CLs in model studies has many advantages, especially when the phase behavior and organization of CL and CL‐containing phospholipid model membranes are the focus of biophysical research.^[9]^

Due to its dimeric nature, CL offers particular structural characteristics amongst the class of phospholipids.^[10]^ Each of the two phosphate groups can carry one negative charge. CL exhibits two chemically different phosphate moieties, although it has a symmetrical structure and four identical alkyl residues.^[11]^ This is due to the chiral centers in each glycerol group. Alterations in the lipid packing can lead to structural changes and thus instabilities in the membrane shape.[^5^, ^12^, ^13^] The ionization behavior is affected by environmental conditions and mainly by the pH value.^[14]^ It was observed that changes in the pH mainly affected the packing behavior of CL‐containing membranes.^[12]^ Controlling the environmental modifications enables the targeted study of these changes. In the past, various research activities have addressed the ionization behavior of CL and some of the results turned out to be contradictory. Instancing, a model with two significantly different pKa values was assumed over several years.^[15]^ Later investigations raised justified doubts about the opinion that both phosphate groups acted like strong dibasic acids.[^16^, ^17^] The pKa values of CL were determined to be around the first pKa of phosphatidic acid and one unit larger. A fundamental understanding of the biological function of CL implies further studies on its acidic properties. CL‐containing membranes experienced the most significant modifications upon pH changes.^[12]^ In fact, pH induced changes in the lipid packing led to instabilities in the membrane curvature.[^17^, ^18^] In addition, electrostatic interactions with proteins and protein binding are also affected by changes in head group charge.^[19]^ It is worth to mention that the head group ionization is unaffected by either the loss of the central glycerol hydroxyl group or of an alkyl chain.^[17]^

The influence of environmental conditions on membranes can be successfully studied by using a basic and well‐defined model membrane system – Langmuir monolayers. The method takes advantage of the consideration that a biological membrane is composed of two weakly coupled monolayers. As the lipid monolayer is formed at an air/water interface, it is feasible to study interactions and properties of single lipids and lipid mixtures as well as monolayer/subphase interactions.[^20^, ^21^] Due to its amphiphilic nature the lipids self‐assemble at the air/water interface and can be studied by highly sophisticated surface‐sensitive methods.[^22^, ^23^, ^24^] The first and simplest technique for identifying the basic phase states and the transitions between them is the recording of pressure‐area (π–A) isotherms.[^23^, ^25^, ^26^] After spreading the lipid solution onto an aqueous subphase, a monolayer is formed that can be compressed by one or two movable barriers to increase the packing density. Thereby, phase transitions can be triggered and recorded. At very large molecular areas, the monolayer is initially in a gaseous state. Depending on the chemical structure of the lipid, either the so‐called liquid‐expanded (LE) phase, characterized by chains in a disordered (*gauche*) state, or a liquid‐condensed (LC) phase is formed by compression. When the LE phase is formed first, a plateau region characterizes the first‐order phase transition to an LC phase during further compression. In the course of the plateau, the two phases coexist. The condensed phase is identified by a less compressible film and by chains that are in the ordered (*all‐trans*) state. Langmuir monolayers generally provide good stability, although the condensed state is a metastable one in most cases.[^20^, ^21^, ^25^, ^26^, ^27^] The structure of a condensed phase can be determined by grazing incidence X‐ray diffraction (GIXD).[^23^, ^28^, ^29^, ^30^, ^31^]

The present work addresses the investigation of the pH dependent properties of 1,1’,2,2’‐tetramyristoyl cardiolipin (TMCL) monolayers. For this purpose, the dissociation behavior of the TMCL head groups was probed by using subphases with different pH values in the range from 3 to 9. An increase of the pH leads to a higher charge density in the monolayers since the phospholipid head groups are protonated at low pH and deprotonated at high pH. Total reflection X‐ray fluorescence is the method of choice because it allows quantitative determination of the amount of counter‐ions and therefore the exact charge density of the monolayer.[^32^, ^33^, ^34^, ^35^] When quantitatively analyzing TRXF data, it is important to avoid competition between different cations.^[36]^ Therefore, the used subphases should contain only one type of cation. For the experiments, the recording of surface pressure versus molecular area isotherms was coupled with GIXD and TRXF. The multimodal measurements were performed at the PETRA III synchrotron facility, DESY (Hamburg, Germany), which allowed a more detailed study of TMCL at the air/water interface.

## Results and Discussion

**Figure 1:**
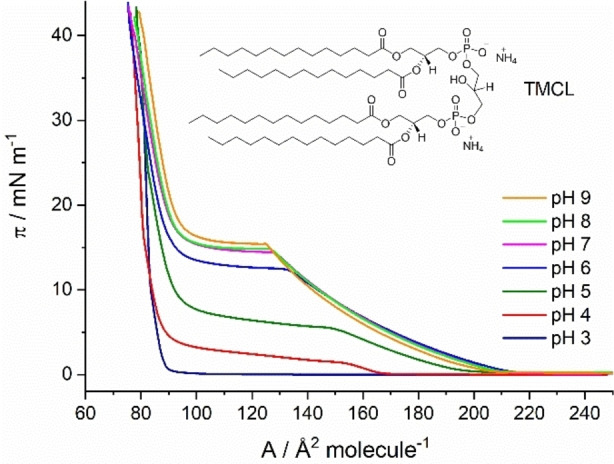
Surface pressure (π) ‐ molecular area (A) isotherms of TMCL monolayers on 1 mM Cs subphases with 0.1 mM EDTA (to bind divalent cations). The measurements were carried out at different pH values and a temperature of 20 °C. With increasing pH, the transition from the LE to the LC phase shifts to higher pressure values.

First, pressure‐area isotherms were measured on subphases with different pH values. The isotherm on the pH 3 subphase indicates that the monolayer does not form a liquid‐expanded (LE) phase (Figure 1). Instead, the gas‐analogous state transforms directly to a liquid‐condensed (LC) state (re‐sublimation). This occurs at practically zero lateral pressure. At higher pH values, the typical first‐order phase transition between LE and LC can be seen. Attention should be paid to an inclined plateau region which is particularly pronounced at pH 4 and 5. One explanation could be the coexistence of TMCL molecules with different ionization states. For the other pH values, the deviation of the plateau from horizontal is much smaller. This could be a sign of the equivalence of the ionization state of most of the molecules in the monolayer. Generally, the phase transition pressure increases and the length of the LE‐LC plateau decreases with rising pH. This is in accordance with increasing deprotonation of the CL head groups.

The lift‐off area shifts to higher values with increasing pH up to pH 6. Between pH 6 and pH 9, the lift‐off area is almost constant (∼206 Å^2^ molecule^−1^). Between pH 7 and pH 9, the LE‐LC phase transition occurs at nearly the same pressures, and only a marginal pressure increase is observed. This could indicate that the head groups are already virtually completely deprotonated at pH 7. The parameters of the LE‐LC phase transitions are summarized in Table 1.

**Table 1:** Parameters of the LE‐LC phase transitions of TMCL (π_start_, π_end_, Δπ, A_LE_, A_LC_, ΔA) obtained by π‐A isotherms. π_start_ describes the starting and π_end_ the ending pressure value of the plateau region. Correspondingly, A_LE_ defines the area in the LE state at π_start_ and A_LC_ the area in the LC state at π_end_ of the plateau region.

pH	π_start_	π_end_	Δπ	A_LE_	A_LC_	ΔA
	–mN m^−1^—			–Å^2^ molecule^−1^—		
3	0	0.3	0.3	–	88	–
4	1.4	3.7	3.3	156	88	68
5	5.4	7.5	2.1	150	91	59
6	12.5	13.4	0.9	131	90	41
7	14.4	15.1	0.7	127	91	36
8	14.7	15.4	0.7	126	91	35
9	15.5	16.3	0.8	124	93	31

In order to shed more light on the protonation degree of TMCL at different pH values, the π–A isotherm measurements were combined with TRXF. The determination of the ionization degree of the phosphate head groups is based on the concentration of the corresponding counter ions in the electrical double layer (EDL) near a charged surface. Cesium is not biologically relevant, but was chosen as the most suitable chemical element for such experiments because it provides a significant fluorescence signal in the accessible energy range of the synchrotron beamline P08 at PETRA III. The TRXF signal intensity is directly proportional to the Cs^+^ concentration in the EDL. GIXD can be simultaneously measured and provides information about the structure of condensed monolayer phases.^[37]^

To avoid influences of competing divalent calcium ions on the interactions of the TMCL head groups with cesium ions from the subphase, 0.1 mM of the complexing agent ethylenediaminetetraacetic acid (EDTA) was added to the 1 mM cesium subphases. EDTA binds divalent ions and therefore only monovalent ions are allowed to form the EDL at the negatively charged surface. For deeper insights into the impact of calcium on negatively charged phospholipid monolayers we refer to our previous publication.^[36]^

During the experiment, the attraction of positively charged ions from the subphase to the negatively charged phosphate groups of the monolayer leads to the formation of an EDL. For quantification, it is essential that only one cation species interacts with the CL monolayer. The amount of ions in the EDL is directly proportional to the ionization degree of the head groups. For charge determination, the corresponding fluorescence line intensities of TMCL were compared with those of the reference substance behenyl sulfate (BS).

**Figure 2:**
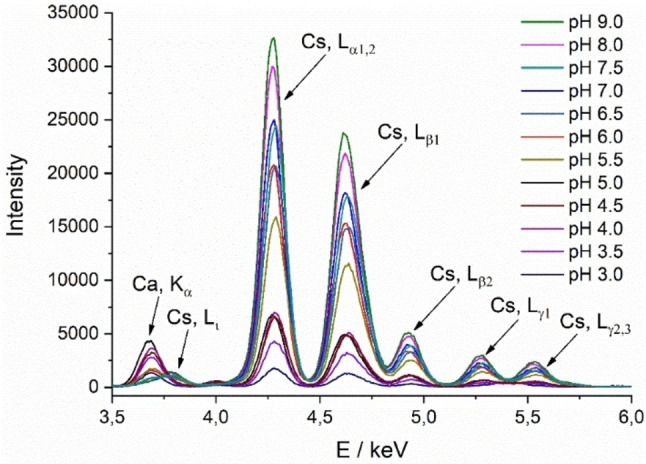
TRXF spectra: The fluorescence intensities of TMCL on 1 mM cesium subphases with 0.1 mM EDTA are shown at different pH values. The spectra of the corresponding bare subphases have been measured before spreading the TMCL monolayer. The line intensities of the cesium bands increase with increasing pH and thus with decreasing protonation degree. At low pH values, competing calcium ions are not chelated by EDTA. The temperature was set to 20 °C.

Figure 2 shows the fluorescence spectra of TMCL at different pH values. The most pronounced Cs lines are L_α1,2_, L_β1_, L_β2_, L_γ1_, and L_γ2,3_ at 4.28, 4.62, 4.94, 5.28, and 5.55 keV, respectively. The low intensities of the cesium bands at pH 3 indicate almost completely protonated CL head groups. With rising pH, the protonation degree decreases and the intensity of the cesium bands increases. At pH 9, the phospholipid head groups should be completely deprotonated. At low pH values, additional calcium lines (K_α_ and K_β_) appear at energies of 3.69 and 4.01 keV. In this case, competing Ca^2+^ ions present as trace amounts in the subphase are not chelated by EDTA.^[36]^ The ability of EDTA to build chelates depends on the pH value, which for Ca^2+^ is recommended to be in the range of pH 5 to 14. The divalent Ca^2+^ is not effectively chelated by EDTA at pH values below 5. When EDTA builds entire complexes with calcium, an additional weak cesium line (L_ι_) becomes visible at 3.80 keV. At low pH, this line is overlain by calcium (Figure S1). Therefore, at low pH, competition between divalent calcium and monovalent cesium ions must be considered for quantitative evaluation of the CL ionization degree.

An established way of determining absolute ion excesses is a calibration procedure utilizing monolayers with known charge density on subphases that contain only one type of counterion. The counterion adsorption to the reference monolayer has to be dominated by unspecific electrostatic attraction and complete charge‐neutralization. Typical reference monolayers are dioctadecyldimethylammonium bromide (DODAB) for the study of anions and behenyl sulfate (BS) for the study of cations.[^32^, ^38^] The standard BS has a known charge density (0.64 C m^−2^) and a permanent charge of −1. BS has no specific interactions with the counter ions and features therefore an adsorption behavior which is only driven by electrostatics. In contrast, the charged phosphate groups of CL exhibit ion specific interactions especially with calcium.^[36]^

The X‐ray fluorescence spectra were analyzed by fitting multiple Gaussian functions to the observed Cs fluorescence lines.^[39]^ Since the intensity I_e_ of the evanescent wave decays exponentially, the thickness of the hydrophobic part z of the monolayer is important for the quantification procedure (I_e_=I_0_ ⋅ exp(−z/Λ) where I_0_ is the maximum electric field intensity, z is the distance from the interface, and Λ is the penetration depth (decay length) of the evanescent wave (in aqueous solutions taken as 7 nm). The attenuation factor for the incoming X‐ray intensity for a distance from the top of the monolayer till the EDL, which is most likely <5 Å thick and near the charged head groups has to be determined for each monolayer separately.^[32]^ The thickness z of the chain region of BS has been determined using z=MW_hch_/(N_A_ ⋅ A ⋅ ρ) with MW_hch_ as the molecular weight of the hydrophobic chain, the molecular area A, determined from isotherm experiments, and the density of the hydrophobic layer ρ multiplied with the Avogadro constant (N_A_). The density value of polyethylene (0.9 g cm^−3^) was taken as that of the hydrophobic layer. With A ∼25 Å^2^, a thickness z=22.8 Å was determined. The attenuation factor is therefore 0.722. The fitted intensity values have to be multiplied by 1.385 before subtracting the background signal from the bare subphase.

The same procedure has to be performed with the TMCL monolayers on the different subphases. In condensed monolayers, the tilt angle of the alkyl chains, which are in *all‐trans* conformation, can be determined by GIXD. The thickness z can be directly estimated using the theoretical length of a stretched chain and the determined tilt angle t with respect to the surface normal. The maximum length of a stretched alkyl chain with n CH_2_ groups is l_max_=(n ⋅ 1.26+1.54) Å.^[40]^ With that value, the thickness of the hydrophobic chain layer is z=l_max_ ⋅ cos(t). The GIXD experiments were carried out at different pressures along the LC phase of the isotherm. Thus, second‐order phase transitions (transition from oblique to rectangular unit cells (obl – L_2_)) and tilting transitions (L_2_ – LS) were also identified. At this point, the transition from a monolayer structure with tilted chains to a structure with non‐tilted chains is well defined and can be precisely determined, whereas the (obl – L_2_) transition pressure can only be estimated due to the lack of data points in the GIXD experiments (larger error bars in Figure 5). Exemplary, the results of the GIXD measurements at pH 6 are shortly described.

**Figure 3:**
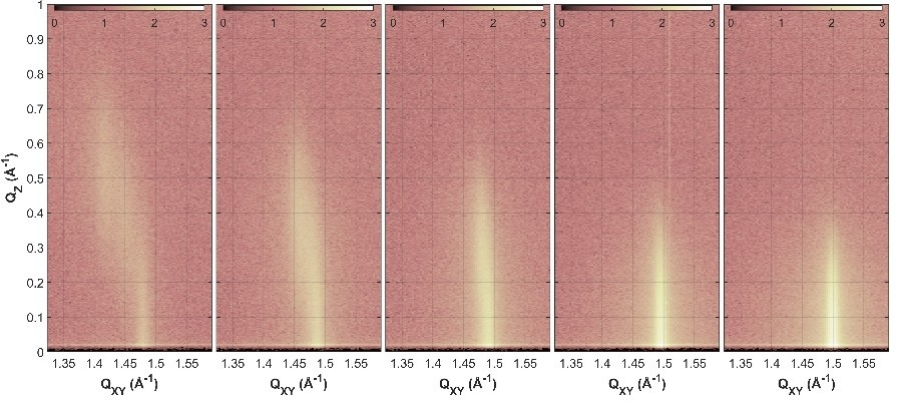
Contour plots of the X‐ray intensities versus the in‐plane (*Q_xy_*) and out‐of‐plane (*Q_z_*) components of the scattering vector of TMCL at pH 6. The GIXD measurements were performed at various pressures along the LC part of the isotherm (from left to right: 14, 22, 26, 30, 35 mN m^−1^). The subphase contained 1 mM Cs and additional 0.1 mM EDTA. The temperature was set to 20 °C.

Figure 3 shows the contour plots of the diffracted X‐ray intensities versus the in‐plane (*Q_xy_*) and out‐of‐plane (*Q_z_*) components of the scattering vector (the corresponding GIXD data and structural parameters are shown in Table S1). At 14 mN m^−1^, three Bragg peaks indicate an oblique structure (obl) with tilted chains (Figure S2). Upon further compression of the monolayer, the tilt angle of the chains is reduced. This leads initially to an orthorhombic (L_2_) phase with NN (nearest neighbor) tilted chains defined by two Bragg peaks. At the tilting transition pressure, the structure changes to a hexagonal packing of non‐tilted chains (hexatic LS phase).^[23]^ The hexatic phase is finally indicated by only one Bragg peak. The hexagonal packing of the chains is known from the literature and is based on a good match of the in‐plane area requirements of the head groups and the four chains of one CL molecule.^[41]^

**Figure 4:**
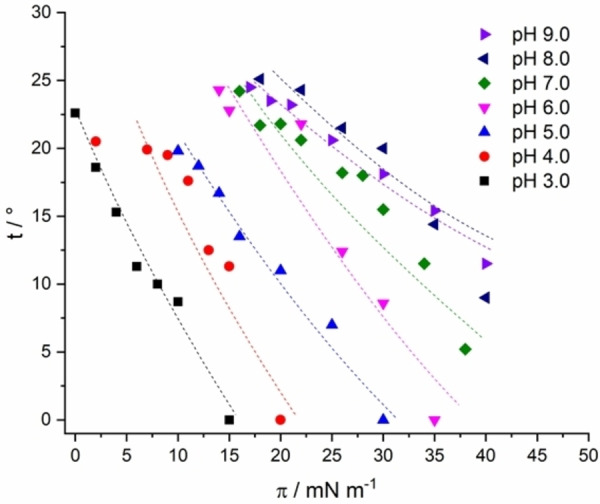
Tilt angle (t) of the alkyl chains versus the surface pressure (π) of TMCL monolayers on subphases with different pH values (indicated) containing 1 mM cesium at 20 °C. An increase in pH leads to a stronger tilt of the chains. The dashed lines are guides for the eyes only.

Comparison of the tilt of the chains at different pH values directs us to Figure 4. Depending on the subphase pH and thus the ionization degree, the alkyl chains are more or less tilted in the LC state. An increase in pH leads to an increase in head group charge and eventually to stronger head group repulsions and therefore to an increased tilting of the chains. The differences in the tilt angle are pronounced at low pH and equalize at high pH, indicating a comparable ionization degree above pH 7.

In order to determine the second‐order tilting phase transition pressure, a constant cross‐sectional area of the chains and an almost linear pressure‐area relationship in the condensed state of the isotherm are assumed.[^23^, ^42^] The tilting transition pressure is yielded by the extrapolation of 1/cos(t) versus the lateral pressure π to 1 (Figure S3). Upon compression of the monolayer, the tilt angle of the chains decreases as well as the lattice distortion. In accordance with a modified Landau theory, the lattice distortion is a linear function of sin^2^(t).[^23^, ^43^] As typical for LS phases, the plot leads to zero distortion at zero tilt (Figure S4). The cross‐sectional area of the hydrocarbon chains in the LC phase remains almost constant during compression (∼20.0 Å^2^). The correlation length increases during compression, indicating a packing of the chains with fewer defects in the LC domains.

**Figure 5:**
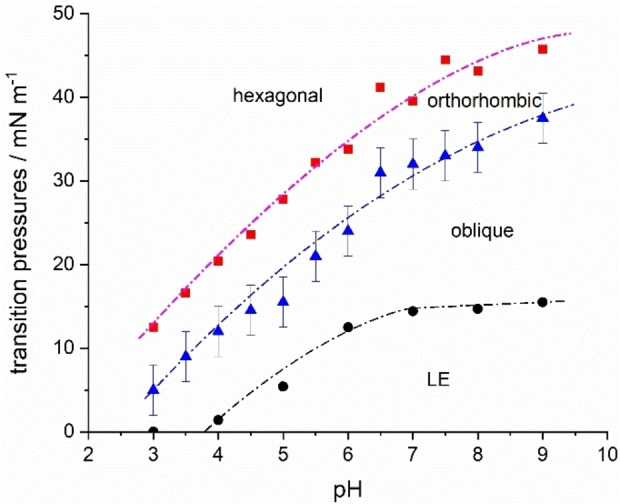
Phase transition pressures of TMCL in dependency on the subphase pH at 20 °C. The LE – LC transition pressures π_start_ (•) were obtained from π‐A isotherms, and the LC transitions oblique to orthorhombic (▴) and tilted to non‐tilted (▪) by GIXD experiments. The dashed‐dotted lines are guide for the eyes only.

In summary, all phase transitions of TMCL at the different pH values are shown in Figure 5. Langmuir isotherm measurements disclosed that the first‐order LE‐LC phase transition is shifted to higher pressures with increasing pH. In comparison, the shift is obvious at lower pH values (pH 3 to 6) and only slight at higher pH values (pH 7 to 9). The small shifts in the higher pH range could indicate almost completely deprotonated head groups above pH 7. Thereby, the results are similar to the observations of Sathappa and Alder, who found that tetraoleoyl cardiolipin (TOCL) is fully ionized at pH 7.5.^[17]^ The GIXD measurements along the LC phase of the TMCL monolayers provided insights into the shift of the second‐order phase transitions to higher pressures with increasing pH (Table S2). In contrast to the LE – LC first‐order transition, both second‐order transition pressures continue to increase with increasing pH, indicating that pH 7 may not be high enough to completely deprotonate the head groups.

**Figure 6:**
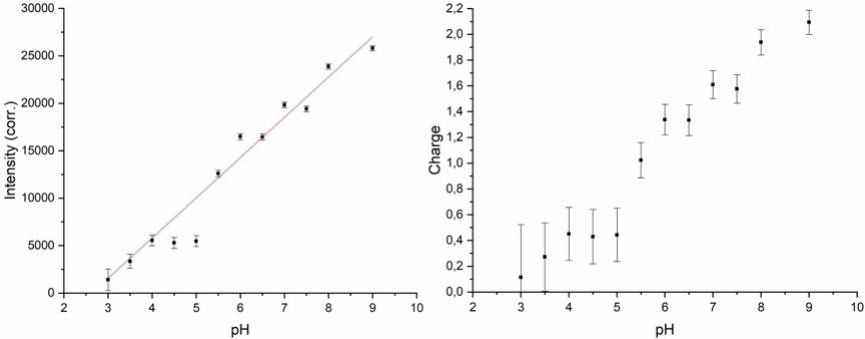
Corrected intensities of the Cs (L_α_) line versus the bulk pH (left) as well as the calculated charge of TMCL versus the bulk pH (right). The measurements were performed at pH values between 3 and 9 and a temperature of 20 °C.

The intensities of the Cs L_α_ lines were used to determine the charge state of TMCL in monolayers on subphases with different bulk pH. The experimentally obtained intensities were corrected for the thickness z of the hydrophobic part as described above. The corrected intensities are plotted vs. the bulk pH (Figure 6, left). Due to a stronger deprotonation of the phosphate groups with increasing pH and the resulting stronger attraction of counterions, the band intensity increases with rising pH. At pH values between 4 and 5, the intensity is quite similar. This is due to competing Ca^2+^ ions, which are not effectively chelated by EDTA at pH values below 5. Above pH 5, the Cs line intensities increase nearly linear with increasing pH.

Based on the values obtained with the behenyl sulfate monolayer, the same smeared curve shape is seen in the plot of the calculated charge of TMCL versus the bulk pH (Figure 6, right). The plot demonstrates that the charge increases rather linear from 0 at pH 3 to 2 at pH 8. At pH 5.5, one phosphate group is charged. Above pH 8, the TMCL head groups are completely deprotonated. The obtained titration curve does not allow the determination of two distinct pKa values, but indicates that the ionization of the two phosphate groups may be influenced by each other. Due to the proximity of these groups, the individual pKa values are shifted to higher respectively lower values. The obtained results demonstrate that the charge density increases continuously over the entire pH range from pH 3 to pH 8.

**Figure 7:**
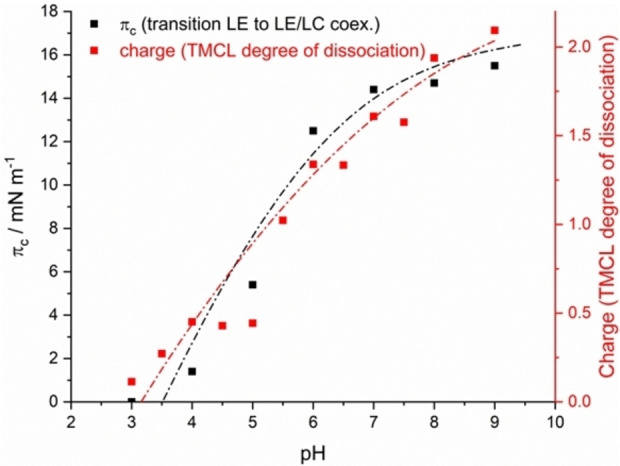
Transition pressures π_c_ obtained by π‐A isotherm measurements versus bulk pH in comparison with the determined charge of TMCL at 20 °C.

Relating the π_c_ values of the π–A isotherm measurements to the charge of TMCL at different pH shows that both methods are complementary (Figure 7). The measurements of the π–A isotherm already give a qualitative idea of the pH dependence of the protonation state of the TMCL head groups. Only the use of TRXF allows quantification of the charge state with specific differences. The most striking difference is the extent of the pH range from fully protonated to completely deprotonated phosphate groups.

## Conclusions

The phospholipid TMCL was studied at various pH values in order to determine the different degrees of protonation of the head groups. For this purpose, the well‐known Langmuir monolayer technique (π–A isotherm measurements) was coupled with TRXF. The π–A isotherm experiments provided information about the pressures of the first‐order phase transitions and revealed that the transition is shifted to higher pressures with increasing pH. The pressure shifts are relatively large at lower pH values (pH 3 to 6) and only marginal at higher pH values (pH 7 to 9). The TRXF studies supported the observations and gave quantitative information about the charge of the TMCL head groups at different pH values. The charge increases with rising pH and the head groups are completely deprotonated above pH 8. The surface charge density is important for many properties of biological membranes. Depending on the ionization state, CL tends to form non‐lamellar phases because of the conical shape (the area requirement of the protonated head groups is smaller than that of the bulky hydrocarbon chain region). However, deprotonated head groups stabilize lamellar phases due to electrostatic repulsion.^[44]^ In literature, there are different models concerning the dissociation behavior of CL head groups. In one model, the pKa values of the phosphate groups differ by several units (2–4 and >8.5) and the head group is a monoanion at physiological pH.^[15]^ In the other model, both phosphate groups have low pKa values (2.5–3.5) and the head group is already in the dianionic form at physiological pH.[^16^, ^45^] Our experiments strongly support the first model but with a continuous course of the charge state versus bulk pH. Obviously, the CL density (highly compressed monolayer or mixtures with other phospholipids) also has a strong influence on the ionization behavior. In the present case, the two phosphate groups influence each other, and continuous ionization was observed over a wide pH range (from 3 to 8). In such a system with high CL packing density, the head groups carry a charge of 1.5 at physiological pH.

GIXD measurements yielded information about the second‐order phase transitions into a non‐tilted state, and the results are perfectly in agreement with those obtained by π–A isotherm measurements.

In summary, this work provides valuable insights into the protonation state of closely packed TMCL in a monolayer at different bulk pH values. In the physiological pH range, the TMCL head groups are already highly deprotonated. The results do not allow for assigning single pKa values to the two phosphate groups. Instead, a mutual influence is observed.

## Experimental Section

### Materials and Monolayer Preparation

The phospholipid 1,1’,2,2’‐tetramyristoyl cardiolipin (TMCL, C14 : 0, purity >99 %) was purchased as an ammonium salt from Avanti Polar Lipids, Alabaster, AL, USA. Chloroform (CHCl_3_, purity >99 %) was obtained from VWR, Vienna, Austria. Cesium hydroxide monohydrate (CsOH, purity ≥90.0 %), EDTA (purity ≥99.9 %) and phosphoric acid (H_3_PO_4_, purity ≥99.9 %) were purchased from Sigma Aldrich GmbH, Taufkirchen, Germany. Boric acid (H_3_BO_3_, purity >99 %) was obtained from Merck KGaA, Darmstadt, Germany, and citric acid monohydrate (C_6_H_8_O_7_×H_2_O, purity ≥99.5 %) from Carl Roth GmbH, Karlsruhe, Germany.

The powdered TMCL was dissolved in chloroform. For the experiments, the solutions were spread to aqueous subphases containing 1 mM cesium using a micro‐syringe. Depending on the desired bulk pH, 1 mM cesium hydroxide was adjusted with citric acid, phosphatidic acid or boric acid. The π–A isotherms were recorded on a computer‐interfaced Langmuir trough (R&K, Potsdam, Germany) equipped with a Wilhelmy balance system. Measurements were started after a waiting period of 10 min to ensure complete evaporation of the solvent. The surface tension of water (σ_w_) was measured with a filter paper Wilhelmy plate, showing a decrease with increasing concentration of amphiphilic molecules in the monolayer at the surface (σ_F_). The surface pressure π=σ_w_–σ_F_ is plotted versus the molecular area A. The temperature was adjusted and stabilized at a constant level using a recirculation cooler. The compression speed of the film was 5 Å^2^ molecule^−1^ min^−1^. All isotherms were measured at least twice to ensure reproducibility.

### Beamline Setup

At beamline P08 (PETRA III), a set of two monochromators (Silicon double crystal (Si111) and Germanium double crystal (Ge311)) was used to receive a monochromatic synchrotron beam. Adjusting the photon energy to 15 keV resulted in a wavelength of 0.827 Å. The Langmuir trough housed in a hermetically sealed container with X‐ray transparent Kapton windows was used for the experiments. The illuminated monolayer surface was approximately 2 mm ×50 mm. In order to remain below the critical angle for total external reflection of water, the incident angle was set to 0.07°. A glass block was located in the subphase under the illuminated area of the monolayer to reduce mechanically excited surface waves.

### TRXF Experiments

TRXF is an element‐specific complementary scattering technique.[^32^, ^34^, ^35^] The fluorescence signal was detected by an Amptek X‐123SDD detector (Amptek, Bedford, United States of America). The detector was positioned parallel to the liquid surface and perpendicular to the photon beam axis in order to keep the Compton scattering at the given polarization of the photons as low as possible. The footprint center of the incident beam was adjusted to be in the middle of the trough and at the same time in the middle of the view angle of the fluorescence detector.^[36]^ In order to reduce background scattering (Ar, K_α,β_) and absorption of low energy photons (P, K_α_=2 keV), the Langmuir trough was purged with Helium (He).

### GIXD Experiments

For the GIXD measurements, the trough was also purged with He to avoid scattering of molecules from the air and to increase the signal‐to‐background ratio. The intensity of the diffracted beam was determined as a function of the vertical scattering angle α_f_ and the horizontal scattering angle 2θ using a Mythen (microstrip system for time resolved experiments) detector (DECTRIS, Baden, Switzerland). The rotation axis of the detector was defined by the center of the beam footprint and an axis perpendicular to the sample surface. In order to limit the in‐plane divergence of the diffracted beam, a Soller collimator (JJ X‐Ray, Denmark) was placed between sample and detector.

Model peaks were fitted to the integrated data by assuming the in‐plane direction (Bragg peaks, Q_xy_) as a Lorentzian and the out‐of‐plane direction as a Gaussian (Bragg rods, Q_z_) function. Finally, structurally relevant information is obtained from the Bragg peak positions or the centers of the Bragg rods. Three Bragg peaks define an oblique lattice, while two peaks describe an orthorhombic one, and one peak finally a hexagonal lattice of non‐tilted lipid alkyl chains.[^23^, ^28^, ^29^, ^31^, ^37^]

## Conflict of interest

The authors declare no conflict of interest.

1

## Supporting information

As a service to our authors and readers, this journal provides supporting information supplied by the authors. Such materials are peer reviewed and may be re‐organized for online delivery, but are not copy‐edited or typeset. Technical support issues arising from supporting information (other than missing files) should be addressed to the authors.

Supporting Information

## Data Availability

The data that support the findings of this study are available in the DESY Publication Database (PUBDB, e‐mail: l.pubdb@desy.de) upon reasonable request.
