# Supplementary material for: The Effect of pH on the Structure and Lateral Organization of Cardiolipin in Langmuir Monolayers
Source: Chemphyschem. 2022 Aug 3;23(19):e202200218. doi: 10.1002/cphc.202200218 (PMC9804817; doi:10.1002/cphc.202200218)
Supplement: Supplementary file 1 — Supporting Information [file CPHC-23-0-s001.pdf]

ChemPhysChem

Supporting Information

The Effect of pH on the Structure and Lateral Organization of Cardiolipin in Langmuir Monolayers

Marina Sturm,* Olof Gutowski, and Gerald Brezesinski

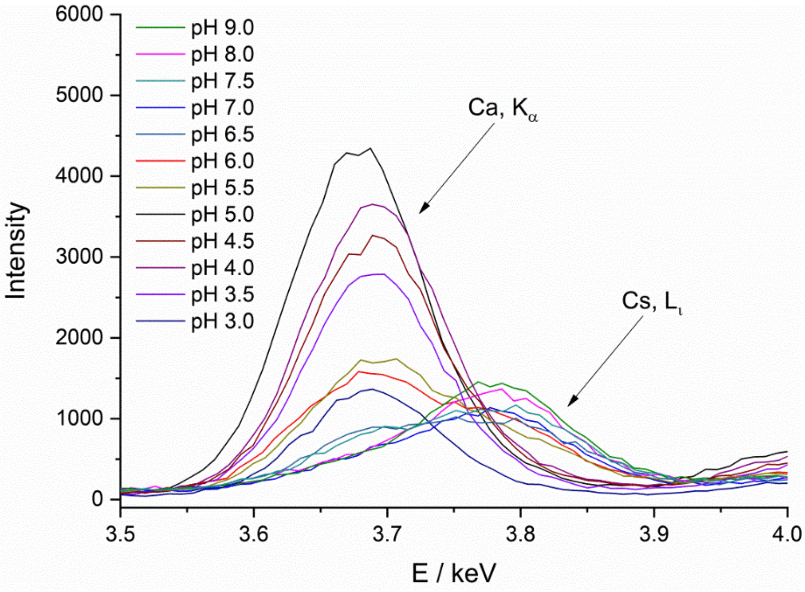

Figure S1. The chart illustrates the ability of EDTA to bind calcium at pH values higher than 5. Below pH 5, the cesium L_{ι} line is completely covered by the calcium K_{α} line. With the binding of calcium by EDTA, the line intensity of calcium becomes weaker (pH 5.5 to pH 6) and the cesium line becomes visible. From pH 6.5 to pH 9, the cesium line is fully visible.

Table S1. GIXD data and structural parameters of TMCL on a subphase containing 1 mM Cs and 0.1 mM EDTA at 20 °C and pH 6. The peak positions (Q_{xy} and Q_z), lattice parameters of the unit cell (a, b, c), distortion (d), tilt angle (t), in-plane area of one chain (A_{xy}), cross-sectional chain area (A_0), and correlation lengths (L_{xy}^1, L_{xy}^2, L_{xy}^3) are presented.

π, mNm^{-1}	Q_{xy}^1, $Å^{-1}$	Q_z^1, $Å^{-1}$	Q_{xy}^2, $Å^{-1}$	Q_z^2, $Å^{-1}$	Q_{xy}^3, $Å^{-1}$	Q_z^3, $Å^{-1}$	a/b/c, Å	d	t °	A_{xy} $Å^2$	A_0 $Å^2$	L_{xy}^1 Å	L_{xy}^2 Å	L_{xy}^3 Å
14	1.479	0.145	1.458	0.438	1.427	0.583	4.896 5.002 5.074	0.0415	23.1	21.6	19.8	350	164	138
15	1.483	0.160	1.463	0.422	1.434	0.582	4.884 4.982 5.050	0.0389	22.8	21.4	19.8	306	118	118
22	1.486	0.136	1.469	0.391	1.456	0.527	4.887 4.931 4.988	0.0236	20.6	21.1	19.7	350	126	186
26	1.491	0	1.482	0.281	-	-	4.906 4.876	0.0081	12.4	20.7	20.2	588	208	--
30	1.495	0	1.493	0.195	-	-	4.862 4.856	0.0018	8.6	20.4	20.2	681	225	--
35	1.500	0	-	-	-	-	4.837	0	0	-	20.3	506	-	-

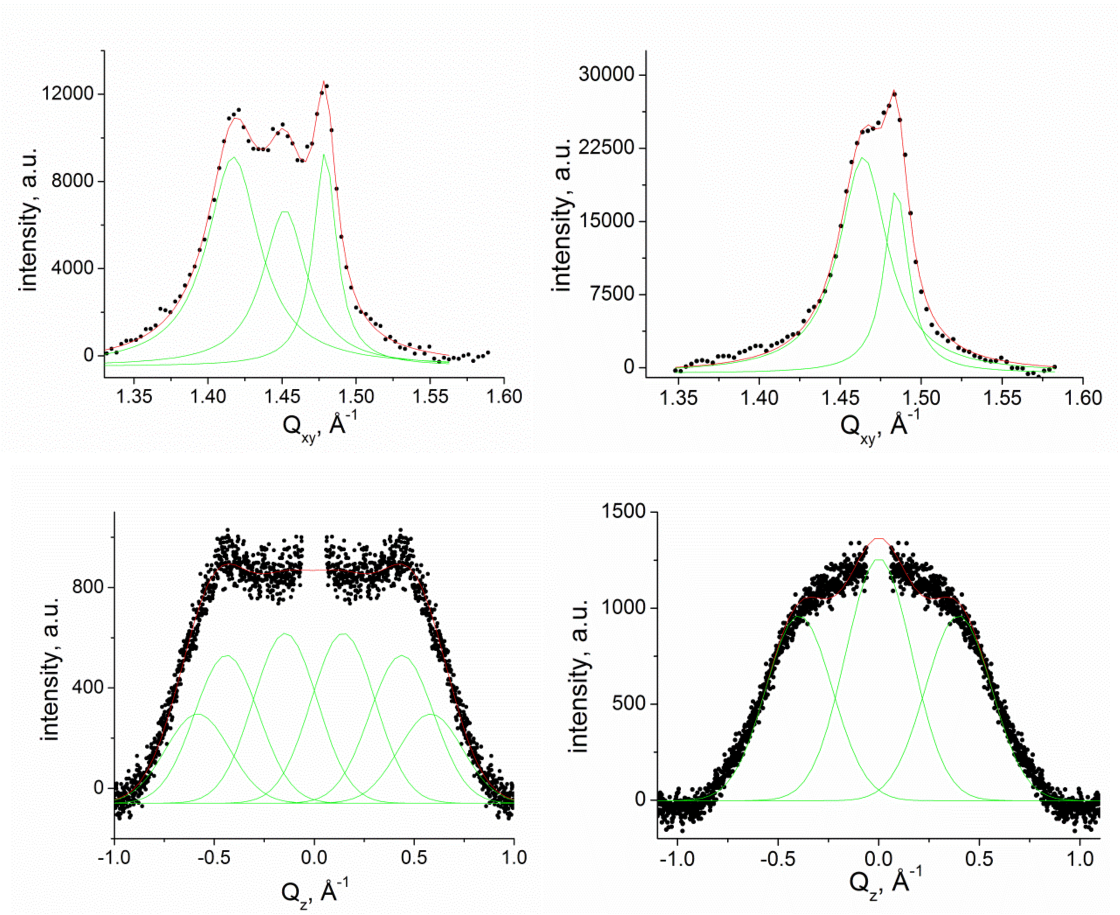

Figure S2. Bragg peaks (top) and Bragg rods (bottom) of TMCL on subphases at pH 6. Left: subphase containing 1 mM Cs^+ and 0.1 mM EDTA, π = 14 mN m^{-1}, oblique phase. Right: subphase containing 1 mM Cs^+ and 0.1 mM EDTA, π = 15 mN m^{-1}, orthorhombic phase L_2.

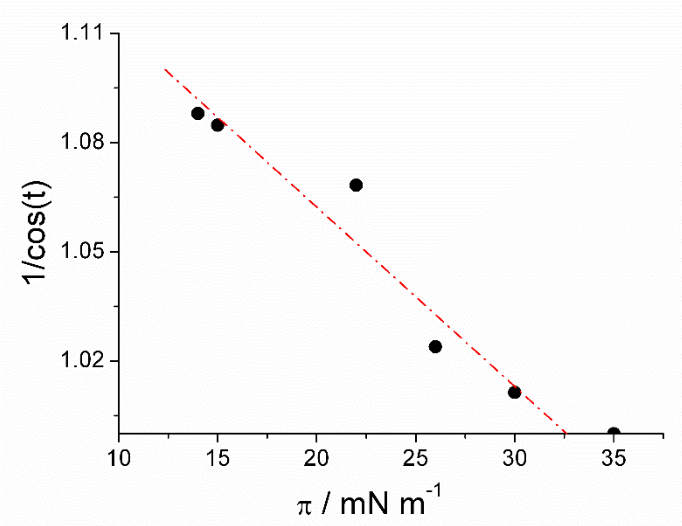

Figure S3. 1/(cos · t) versus the lateral pressure of TMCL monolayers on a subphase containing 1 mM Cs and 0.1 mM EDTA at 20 °C and pH 6. The linear extrapolation toward zero tilt angle (1/(cos · t) = 1) yields the pressure π_{t2} of the tilting transition (see table S2).

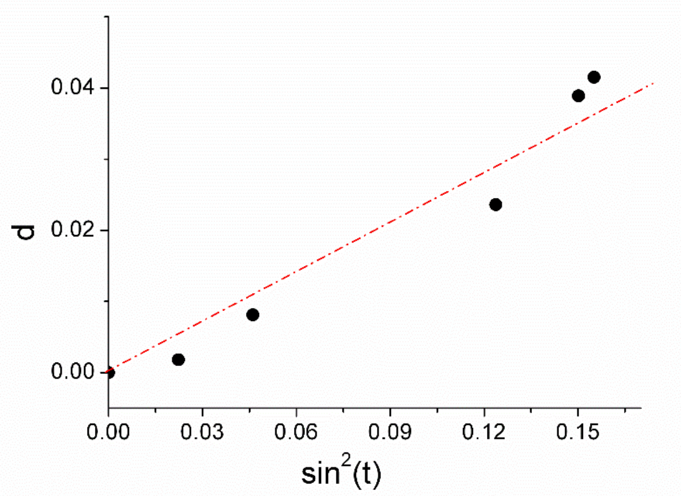

Figure S4. Lattice distortion d versus $\sin^2(t)$ of TMCL monolayers on a subphase containing 1 mM Cs and 0.1 mM EDTA at 20 °C and pH 6.

Table S2. Phase transition pressures (π_{t1}: oblique to orthorhombic (L_2) and π_{t2}: L_2 - LC) obtained by GIXD measurements.

pH	π_{t1} mN m^{-1}	π_{t2} mN m^{-1}
3.0	5.0	12.5
3.5	9.0	16.6
4.0	12.0	20.4
4.5	14.5	23.6
5.0	15.5	27.8
5.5	21.0	32.2
6.0	24.0	32.6
6.5	31.0	41.2
7.0	32.0	39.5
7.5	33.0	44.5
8.0	34.0	43.1
9.0	37.5	45.7
